# Is ischemia the only factor predicting cardiovascular outcomes in all diabetes mellitus patients?

**DOI:** 10.1186/s12933-017-0533-7

**Published:** 2017-04-20

**Authors:** Mark W. Kennedy, Enrico Fabris, Harry Suryapranata, Elvin Kedhi

**Affiliations:** 1 Isala Hartcentrum, Docter van Heesweg 2, Zwolle, The Netherlands; 2 Diagram CRO, Zwolle, The Netherlands

**Keywords:** Diabetes mellitus, Ischemia, Fractional flow reserve, Major adverse cardiac event

## Abstract

Diabetes mellitus (DM) is associated with an excess in cardiovascular morbidity and mortality, and is characterized by increased rates of coronary artery disease. Furthermore, once atherosclerosis is established, this is associated with an increased extent, complexity and a more rapid progression than seen in non-DM patients. Ischemia is the single most important predictor of future hard cardiac events and ischemia correction remains the cornerstone of current revascularization strategies. However recent data suggests that, in DM patients, coronary atherosclerosis despite the absence of ischemia, detected by either invasive or non-invasive methods, may not be associated with the same low risk of future cardiac events as seen in non-DM patients. This review seeks to examine the current evidence supporting an ischemia driven revascularization strategy, and to challenge the notion that ischemia is the only clinically relevant factor in the prediction of cardiovascular outcomes in all-comer DM patients. Specifically, we examine whether in DM patients certain characteristics beyond ischemia, such as microvascular disease, coronary atherosclerosis burden, progression and plaque composition, may need to be considered for a more refined risk stratification in these high-risk patients.

## Background

An estimated 415 million people worldwide have diabetes mellitus (DM), with the prevalence expected to increase by a further 50% by 2050 [1]. DM is associated with an excess in morbidity and mortality [2]. Compared to patients without DM, people with DM are between two and four times more likely to have cardiovascular disease (CVD), with CVD accounting for a large proportion of the excess mortality related to diabetes [3–7]. Indeed, 5-year cardiovascular mortality rates amongst those DM patients without a history of coronary artery disease (CAD) are similar to those of non-DM patients with a history of previous MI, and as such DM is considered a CAD equivalent [8].

Furthermore, once atherosclerosis is established, it is associated with increased rates, extent, complexity and more rapid progression than seen in non-DM patients, with resultant poor outcomes [9–11]. In addition, revascularization outcomes in DM patients treated with percutaneous coronary intervention (PCI) continue to be less favorable than in non-DM patients [12]. Despite refinements in stent design over the past number of years which have reduced rates of target lesion revascularization (TLR), major adverse cardiac events (MACE) and stent thrombosis in non-DM patients, regrettably these improvements have not been transferred to DM patients, particularly those with more complex lesions [12, 13]. Alternatively, DM patients with non-complex lesions appear to have similar outcomes to non-DM patients, and so the earlier identification of these patients may improve outcomes and arrest progression [13]. This is particularly salient as for those DM patients with multi-vessel disease, current evidence favors coronary artery bypass grafting (CABG) as the preferred revascularization modality, which is likely to reflect the more complete revascularization and global protection provided by arterial conduits against rapid atherosclerosis progression in PCI and untreated segments.

The Providing Regional Observations Study Predictors of Events in the Coronary Tree (PROSPECT) study and subsequent sub-study analyses have consistently highlighted DM to be associated with worse outcomes. These worse outcomes in DM patients originated not only from the culprit lesion but also from non-culprit lesions (which were of very mild angiographic severity at baseline). Furthermore, patients with DM were more likely to have at least one non-culprit lesion containing multiple high-risk plaque features shown to correlate with future unanticipated cardiac events [14, 15]. Thus, angiographically mild but otherwise “high risk” lesions in DM patients may not be as quiescent as in non-DM patients and so whilst presently the most appropriate revascularization strategy is based on targeting only those lesions causing ischemia, whether this is the only factor in DM patients is questionable [13, 16–18]. This review seeks to examine the current evidence supporting an ischemia driven revascularization strategy, and to challenge the notion that ischemia is the only clinically relevant factor in the prediction of cardiovascular outcomes in all-comer DM patients. Specifically we examine whether in DM patients, certain characteristics beyond ischemia, such as microvascular disease, coronary atherosclerosis burden, progression and plaque composition, may need to be considered for a more refined risk stratification in these high-risk patients.

## The importance of ischemia

Studies have repeatedly shown that ischemia is the most important predictor of outcomes, with the presence of ischemia being associated with a 12-fold increased risk of future adverse cardiac events (death or non-fatal myocardial infarction) compared to those patients without ischemia [19]. Furthermore, the presence of moderate to severe ischemia is associated with a significantly higher risk of subsequent adverse events including death or myocardial infarction compared with mild or no ischemia [20]. In keeping with this, the Clinical Outcomes Utilizing Revascularization and Aggressive Drug Evaluation (COURAGE) nuclear sub-study showed that those patients with the largest burden of ischemia, as detected by single-photon emission computed tomography (SPECT), derived the greatest benefit from revascularization [21]. Conversely, worsening of ischemia is an independent predictor of death or myocardial infarction [22], underscoring the concept of ischemia driven revascularization, whether by PCI or CABG [23–25]. Specifically in DM patients, it has been shown that incomplete revascularization is associated with substantially worse outcomes, particularly in those patients with a large residual burden of ischemia [26, 27].

## Fractional flow reserve and the assessment of ischemia

In the catheterization laboratory, fractional flow reserve (FFR) has emerged as the gold-standard invasive technique to detect coronary stenoses of sufficient hemodynamic severity to induce myocardial ischemia. Given the poor correlation between angiographic severity and ischemic significance, the use of FFR to reliably detect functionally significant lesions-which may otherwise be left untreated with resultant poor outcomes- seems logical and appealing [28]. Conversely, FFR may also identify those lesions which despite angiographic appearances are not functionally significant and do not require revascularization.

In the DEFER study, intermediate coronary stenoses which were non-ischemic (FFR > 0.75 cut off) had a low risk of future adverse cardiac events; approximately 1% per year and this risk was not decreased by revascularization [29]. Additionally, at 5-year follow-up, non-ischemic lesions continued to have significantly better outcomes compared to those lesions which were FFR <0.75 and had index revascularization (5-year event rate; 3.3 vs 15.7%, p = 0.002). Subsequently, the fractional flow reserve versus angiography in multi-vessel evaluation (FAME) trial, enrolled 1005 patients with multi-vessel CAD who were randomized to undergo PCI with drug-eluting stents guided by angiography alone or guided by FFR assessment of ischemia [30]. Patients assigned to FFR-guided PCI underwent stenting of indicated lesions only if the FFR was ≤0.80. The primary end-point was the rate of death, nonfatal myocardial infarction, and repeat revascularization at 1 year, and was 18.3% in the angiography group versus 13.2% in the FFR group (p = 0.02). In keeping with the DEFER study, those lesions which were found to be non-ischemic, had excellent outcomes with medical therapy alone; 2-year rate of myocardial infarction (MI) 0.2, 1.9% revascularization rate. Thus, based on these studies, limiting PCI to only ischemia inducing lesions resulted in significantly better results and deferred revascularization of non-ischemic lesions was associated with excellent outcomes, in addition to significant cost savings [31].

Moreover, similar to non-invasive studies, the FAME II study has shown that those lesions which are ischemia inducing and which are not revascularized, have substantially higher adverse event rates compared to similar angiographic lesions which are non-ischemic, whereas revascularization reduces this risk [32]. The combination of these studies have shaped current guidelines, recommending FFR to identify the hemodynamic relevance of intermediate coronary lesions and deferred revascularization of those lesions FFR >0.80 [25]. Unfortunately, the proportion of patients with DM included in the large FFR studies to date has been low, ranging from 10.8 to 27%, and thus the concept of FFR guided revascularization in a population of only DM patients has perhaps not been completely proven.

## FFR and diabetes mellitus

Diabetes mellitus is characterized by insulin resistance and chronic hyperglycemia, and concerns regarding increased vascular resistance and reduced vasodilative capacity due to chronic hyperglycemia have been raised [33]. Whilst, the reliability of FFR in DM patients is now accepted, more recently data has emerged suggesting that FFR and specifically the deferral of revascularization based upon a FFR >0.80 in DM patients may not be associated with the same low risk of adverse events as seen in non-DM patients [34].

**Fig. 1:**
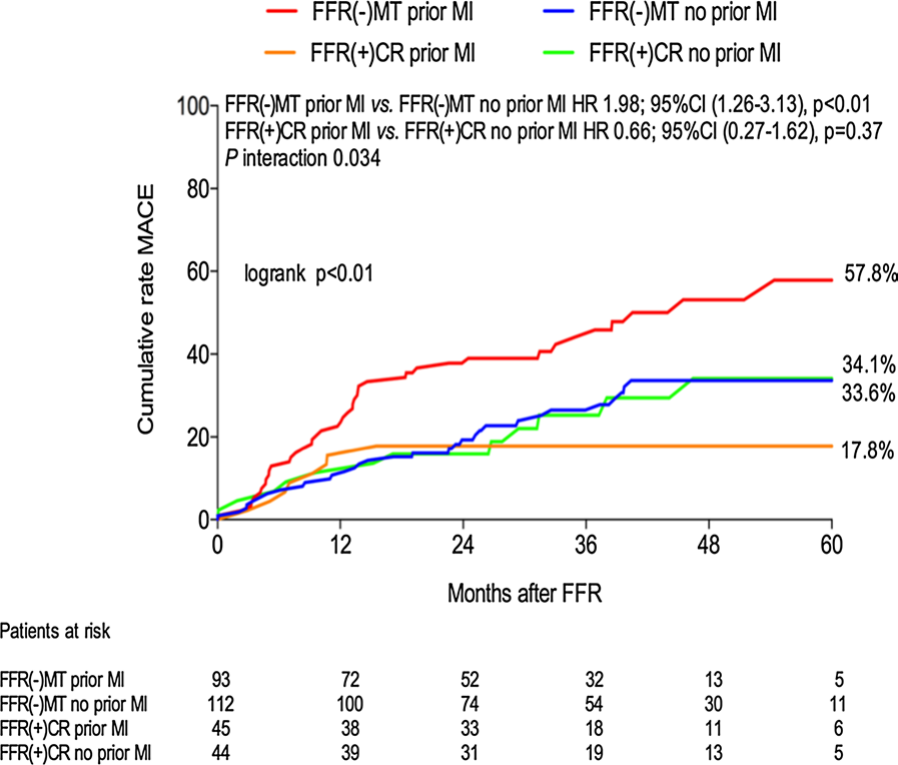
Time-to-event estimates for MACE, in FFR(−)MT and FFR(+)CR groups according to presence or absence of prior MI. *MACE* major adverse cardiac event; *MI* myocardial infarction; *FFR* fractional flow reserve; *FFR*(−)*MT* the group of patients with ≥1 FFR negative (FFR > 0.80) lesion(s) which underwent medical therapy; *FFR*(+)*CR* the group of patients with all lesions FFR positive (FFR ≤ 0.80) and had complete revascularization; *HR* adjusted hazard ratio (Reproduced with permission from Kennedy et al. [35])

**Fig. 2:**
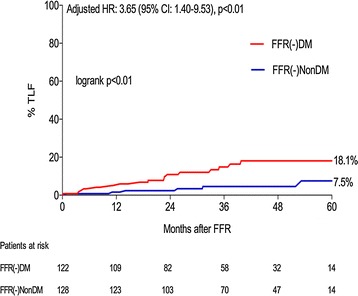
Time-to-event estimates for target lesion failure according to FFR(−)DM and FFR(−)NonDM groups. *TLF* target lesion failure; *CI* confidence interval; *HR* hazard ratio (adjusted for age); *FFR* fractional flow reserve; *FFR*(−)*DM* the group of DM patients with FFR negative (FFR > 0.80) lesions; *FFR*(−)*NonDM* the group of Non-DM patients with FFR negative (FFR > 0.80) lesions (Reproduced with permission from Kennedy et al. [36])

Our group have recently shown that compared to those DM patients who undergo complete revascularization, DM patients with ≥1 remaining FFR negative (>0.80) lesion, have a significantly higher incidence of MACE, a composite of death/MI, rehospitalization for acute coronary syndrome (ACS) and TLR, HR 2.01 (95% CI 1.21–3.33, p < 0.01) [35]. Furthermore, significant clustering of MACE events in those DM patients with a previous MI carrying FFR negative lesions was noted, whereas those patients without a previous MI had much more benign outcomes (Fig. 1). In a separate study, comparing deferred revascularization based upon a FFR >0.80 in 122 DM patients and 128 non-DM patients, DM patients had significantly higher rates of target lesion failure (TLF), HR 3.65 (95% CI 1.40–9.53, p < 0.01), with significantly higher rates of TLR and a clear trend towards a higher incidence of target vessel MI (Fig. 2) [36]. Conversely in non-DM patients, deferred revascularization appeared to be as safe as reported in prior studies, with low rates of future target vessel MI and TLR. Recently, Liu et al. [37] have confirmed these findings and have shown that amongst those patients with an FFR >0.85, diabetics had a more than two-fold higher risk of death and MI than non-diabetics, HR 2.20 (95% CI 1.19–4.01, p = 0.015). In addition, this study also reported that among non-diabetic patients with deferred PCI based upon a FFR >0.80, higher FFR values (closer to 1.0) were associated with lower rates of death, MI and revascularization. However, in DM patients with deferred revascularization, FFR values were unable to differentiate the risk of cardiovascular events, a finding we have recently confirmed, showing that FFR values do not predict future deferred lesion failure in FFR negative lesions in DM patients. Moreover, using multi-variate analysis, the only independent predictors of lesion failure identified were insulin requiring DM and a history of prior revascularization, both conditions being marked by a more rapid atherosclerosis progression [38].

Thus, in all of these studies, deferred revascularization in DM patients based upon the absence of FFR detected ischemia does not appear to be as safe as in non-DM patients. Several possible mechanisms, such as an increased prevalence of microvascular dysfunction, more aggressive atherosclerosis progression, an increased burden of disease and a more active and high-risk plaque composition may contribute to this elevated risk of adverse cardiac events despite the absence of ischemia.

## Complementary hemodynamic assessments and microvascular disease

Whilst FFR provides assessment of epicardial coronary stenosis severity and lesion-level ischemia, clinical events occur even in patients with FFR >0.80, with one possible explanation owing to abnormalities in microvasculature. Coronary flow reserve (CFR) and the index of microcirculatory resistance (IMR) may provide additional complementary information in such situations. Indeed, significant discordance, ranging from 27 to 40%, has been described between FFR and CFR measurements [39, 40]. In a study by Meuwissen et al. [41] in patients undergoing combined FFR and CFR assessment, approximately 10% of intermediate lesions when assessed as FFR non-ischemic, have an abnormal CFR defined as <2.0, a finding confirmed by others. Recently, van de Hoef et al. [42] have shown that in those patients in whom a FFR >0.80 is associated with an abnormal CFR (<2.0), the clinical outcomes are significantly worse than in patients with intact microcirculation.

## Microvascular disease and DM

Data from prior studies which have assessed the microcirculatory function in patients with and without DM, have shown that patients with DM have substantially altered microvascular function and even amongst those DM patients without known CAD, the presence of an abnormal CFR is associated with poor outcomes, comparable to non-DM patients with known CAD [43, 44]. Furthermore, it has been demonstrated that in diabetic patients without obstructive CAD, coronary microvascular function is substantially more impaired than in non-DM patients when matched for traditional cardiovascular risk factors [45]. Finally, in the The Prediction of CK-MB RElease During Successful Stenting Correlating with Indicators of Microvascular ObstruCTion (PREDICT) trial, despite similar pre-PCI FFR values, DM patients after PCI, had significantly lower CFR measurements indicative of greater microvascular dysfunction [46]. It has been postulated that this microvascular dysfunction promotes the process of atherosclerosis. Indeed, this dysfunction is substantially worse in patients with poorer glycaemic control and may contribute to the poorer outcomes seen in such patients [47].

**Fig. 3:**
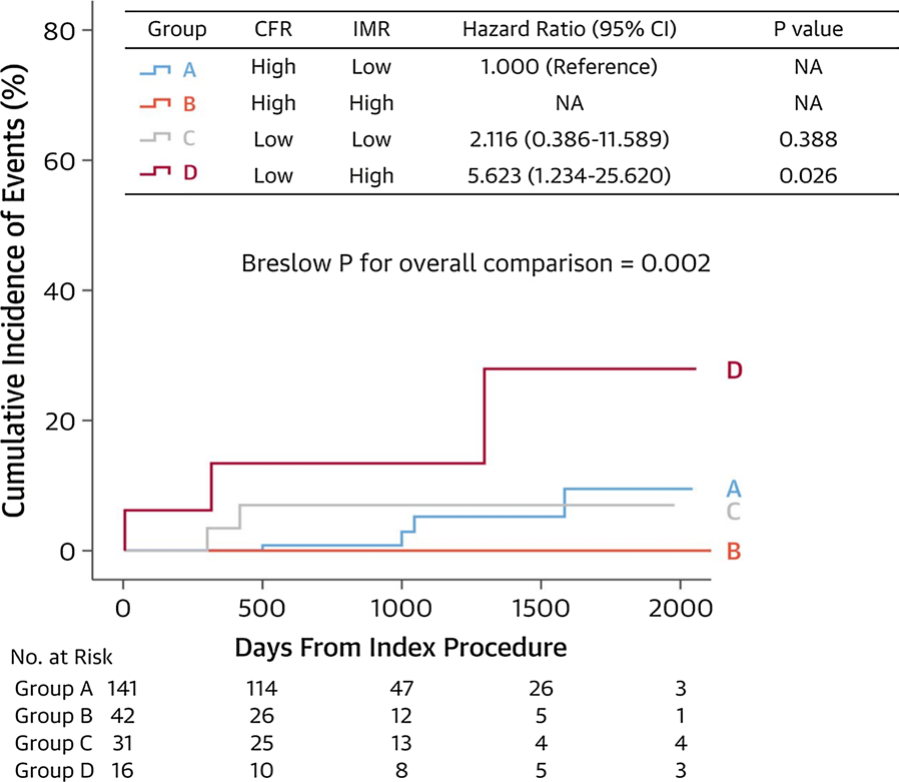
Clinical outcomes according to patterns of microvascular status defined according to CFR and IMR among patients with high FFR. The cumulative incidence of patient-oriented composite outcomes was compared among four groups divided according to CFR and IMR. All IMR values were adjusted with Yong’s formula (IMR_corr_). *NA* not available; *CFR* coronary flow reserve; *IMR* index of microvascular resistance; *CI* confidence interval (Reproduced with permission from Lee et al. [48])

Recently Lee et al. [48] examined the clinical, angiographic, and hemodynamic characteristics of patients with high FFR (>0.80) and evaluated the prognostic implications of abnormal CFR and IMR in these patients. Despite similar clinical and angiographic characteristics, including similar Gensini and SYNTAX scores (to quantify patients’ macrovascular disease burden), patients with a high FFR, a low CFR and a high IMR had a significantly higher adverse event rate during follow-up compared with those patients with intact microcirculation, HR: 5.623 (95% CI 1.234–25.620; p = 0.026) (Fig. 3). Moreover, in a multivariate model comprising those patients with a high FFR, low CFR and high IMR, DM was identified as an independent predictor of adverse events, HR 2.71 (95% CI 1.05–7.02, p = 0.04). Conversely, those patients with a high FFR and normal microvascular function (high CFR, low IMR) had excellent outcomes. Thus, based upon this study, abnormal microvascular function may in part explain the worse outcomes in DM patients despite the absence of FFR detected ischemia, as has been recently described in several studies [35–37]. Whether the addition of complementary hemodynamic assessments in DM patients with negative FFR assessments, may result in a more accurate deferred revascularization needs to be studied in larger dedicated studies and the development of repeatable methods of absolute coronary flow measurement may finally help to provide a better understanding of coronary microcirculation [49].

## Coronary atherosclerosis progression

Coronary atherosclerosis is a generalized disease and the natural history of CAD is that of a progressive condition, thus initially non-obstructive and non-ischemia producing lesions can over time progress to become high-grade stenoses, resulting in cardiac events. Several studies have attempted to assess the impact of atherosclerosis progression on future events. Glaser et al. [50] reported that 6% of initially non-culprit coronary lesions will have clinical plaque progression requiring non–target-lesion PCI by 1 year. Chacko et al. [51] provided 5-year follow-up data from the SIRIUS (Sirolimus-Eluting Stent in De Novo Native Coronary Lesions) trial, and have shown that events attributed to the non-target vessel are frequent and accounted for the majority of all adverse outcomes, with almost 25% of patients suffering an event related to disease progression during the 5-year follow-up period in both the sirolimus and bare metal stent groups. Finally, the contribution of atherosclerosis progression on future events may be even higher; based upon the results of the BASKET-PRO study, 40% of all events at 5 years and almost 40% of all new perfusion defects in patients without events were as a result of disease progression in non-target areas [52].

## Atherosclerosis progression and DM

DM is associated with even more unremitting and rapidly progressive atherosclerosis progression which may be as a result of numerous factors; hyperglycemia induced endothelial dysfunction, increased platelet aggregation, and plaque instability. Additionally when these processes are combined with other traditional risk factors, a synergistic effect occurs which significantly accelerates atherosclerosis progression. In the Prevention of Restenosis with Tranilast and its Outcomes (PRESTO) trial, diabetic patients had a 33% increase over non-diabetic patients in new lesion formation over a nine month follow up [53]. In the SWISSI II study, despite comprehensive cardiovascular risk factor intervention, DM was identified as the strongest predictor of progressive coronary artery disease, OR 19.01, p = 0.03 [54]. Finally, in the Diabetes and Sirolimus Eluting Stent (DIABETES) study, at 2-year follow-up, 50% of repeat revascularizations were as a result of progression in a vessel or segment remote and different from the one previously treated, a finding which has been confirmed by others [55, 56]. Indeed, the aforementioned PROSPECT study, which assessed the natural history of atherosclerosis in patients presenting with ACS has highlighted this very fact, with the majority of subsequent adverse cardiac events, particularly in DM patients arising from so-called non-culprit lesions which given their angiographic appearance (mean diameter stenosis 36.2 [31.1–44.2]) were presumably non-ischemic at baseline [14].

**Fig. 4:**
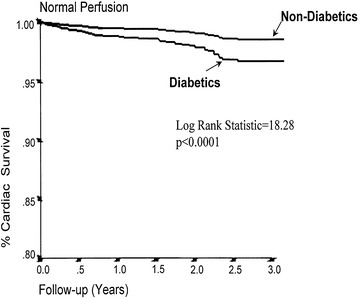
Kaplan-Meier survival curves comparing the subset of diabetic and nondiabetic patients with normal stress MPI results. *MPI* myocardial perfusion imaging (Reproduced with permission from Giri et al. [58])

This more rapid progression may in part explain the findings of the recent studies which have shown worse outcomes in DM patients despite the absence of ischemia. Furthermore, it is recognized that lesions which are angiographically significant are known to progress faster than milder lesions [57]. Thus, despite the absence of ischemia, an “angiographically significant but hemodynamically non-significant” lesion in the setting of rapid atherosclerosis progression in DM, may not be insignificant. Indeed, Giri et al. [58] have shown in a study of 4755 patients undergoing SPECT myocardial perfusion imaging (MPI), of which 929 were diabetic, that survival during the first 2 years of follow-up was identical in the patients with normal MPI results, irrespective of their diabetic status. However rates increased rapidly after 2 years in diabetics but not in non-diabetics (Fig. 4). Thus, the absence of ischemia, assessed either by invasive (FFR) or non-invasive (SPECT) methods in DM patients, does not appear to have the same “warranty” as in non-DM patients.

## Coronary artery disease burden and DM

Compared to non-DM patients, DM is associated with a higher incidence of coronary artery calcium (CAC), an anatomic marker of increased coronary artery disease burden. Furthermore, non-invasive studies combining computerized tomography (CT) CAC scoring and a functional assessment of myocardial ischemia in the same patient, have shown that atherosclerotic burden despite normal ischemia studies predicts adverse cardiac events. Thus, there exists a strong linear relationship between increasing CAC scores and future adverse cardiac events, with a CAC score >400 being a significant predictor, HR 3.55 (95% CI 1.78–7.09; p < 0.001). Alternatively, those patients with normal perfusion and without CAC have excellent outcomes.

The Multi-Ethnic Study of Atherosclerosis (MESA) in which 6814 participants without a prior history of CAD underwent CT assessment to assess the incidence of CAC, showed that compared to non-DM patients, DM patients have double the incidence of CAC presence, RR 1.9 (95% CI 1.4–2.4, p < 0.01). Furthermore, DM was identified as the strongest risk factor for CAC progression, HR 26.8 (95% CI 19.5–34.2, p < 0.001). Conversely, 38% of DM patients had no CAC, and the absence of CAC was associated with a low annual rate (<1%) of CHD events (7). Recently, Blanke et al. [59] published 5-year follow up of the prognostic utility of coronary CT angiography in patients with DM from the CONFIRM (Coronary CT Angiography Evaluation for Clinical Outcomes: An International Multicenter) registry. In this study, 1823 DM were propensity-matched to 1823 patients without DM. In the absence of CAD, DM patients had similar outcomes to non-DM patients, HR 1.32 (95% CI 0.78–2.24; p = 0.30). However, strikingly patients with DM and non-obstructive (diameter stenosis 1–49%) and thus highly likely non-ischemic CAD, had significantly worse outcomes than non-DM patients with obstructive disease (diameter stenosis > 50%), both in terms of all-cause mortality, [HR 2.09 (95% CI 1.43–3.06, p < 0.001)] and MACE [HR 5.12 (95% CI 2.95–8.88, p < 0.001)]. These landmark findings strongly support the concept that DM is associated with therapy refractive, rapidly progressive coronary atherosclerosis and further supports the possibility that such progression may be as a result of significant differences in plaque composition between DM and non-DM patients, as described in the PROSPECT study [11, 14, 15].

## DM and plaque composition

Marso et al. [15] in a sub-study analysis from the PROSPECT study, have shown that DM patients have a significantly different composition and character of atherosclerosis than non-DM patients. Using gray-scale and radio-frequency intravascular ultrasound, non-culprit lesions (NCL) in DM patients were noted to be significantly longer, had a greater plaque burden, a smaller lumen area, and had a greater necrotic core and a larger calcium content. Additionally, necrotic core and calcification were significantly greater in the NCL’s of those DM patients with future MACE compared to DM patients who did not have subsequent event. Furthermore, the use of insulin therapy was also noted to be associated with a significantly higher incidence of NCL-MACE [14].

**Fig. 5:**
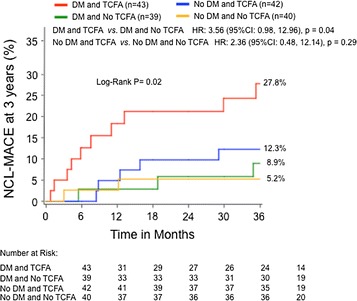
Time-to-event estimates of non-culprit lesion-related MACE, according to the presence of diabetes and/or a TCFA at 3 years in the propensity matched groups of patients with and without diabetes. *DM* diabetes mellitus. *TCFA* thin-cap fibroatheroma. *HR* hazard ratio. *CI* confidence interval. *NCL* non-culprit lesion. *MACE* major adverse cardiac events (Reproduced with permission from Kedhi et al. [60])

In an additional analysis from the PROSPECT study, Kedhi et al. [60] analyzed the incidence of NCL-MACE in two propensity-matched groups according to the presence of DM and thin cap fibroatheroma (TCFA). In this study, among DM patients, the presence of ≥1 TCFA was associated with higher NCL-MACE at 3 years (27.8 vs. 8.9% in patients without a TCFA, HR: 3.56; 95% CI 0.98–12.96; p = 0.04). Alternatively, DM patients without a TCFA had a similar 3-year rate of NCL-MACE as patients without DM (8.9 vs. 8.9%; HR:1.09; 95% CI 0.27–4.41; p = 0.90) (Fig. 5). Thus based upon this study, there would appear to be a symbiotic relationship between vulnerable plaque and DM, which results in excessive adverse outcomes which does not occur to the same degree in non-DM patients with similar plaque.

Moreover, studies have shown that a longer duration of DM and poorer glycemic control are associated with a higher prevalence of TCFA [61, 62]. Since DM not only promotes atherosclerosis progression, the greater oxidative stress and hyperglycemia associated with this condition also favors plaque instability and degradation [63, 64]. Given the higher prevalence of TCFA in DM patients, this may account for the observed elevated risk despite an apparent absence of ischemia. The Impact of optical coherence tomography (OCT) detected TCFA on major adverse events derived from non-ischemic (FFR negative) atherosclerotic lesions in DM patients is currently being studied in the COMBINE study [65]. In this prospective multi-center, study, DM patients with FFR-negative lesions are clinically followed after index OCT assessment and compared for major adverse events based on presence or absence of TCFA. Interestingly, both groups will be compared with a third group of patients with similar angiographic lesions at baseline which were FFR positive and therefore underwent index revascularization. This study will shed important light onto the impact of untreated TCFA in FFR negative lesions and may help to explain the poorer outcomes observed in these non-ischemic lesions.

Finally, in response to progressive atherosclerosis, negative remodeling occurs more frequently in DM patients compared to the typical positive remodeling seen in non-DM patients [66]. This vessel shrinkage and inability to overcome continued intimal hyperplasia may also explain why FFR and other ischemic tests do not appear to carry the same warranty. Moreover, negative remodeling has been shown to be a marker of more advanced atherosclerosis and more abundant TCFA distribution which may also contribute to the reduced guarantee [67, 68].

## Conclusions

DM patients have more rapidly progressive coronary atherosclerosis, a higher degree of microvascular disease, a larger burden of coronary plaque and a significantly different composition of atherosclerosis compared to non-DM patients. Recent evidence has shown that the absence of ischemia, detected by either non-invasive or invasive methods, may not carry the same “warranty” as in non-DM patients. This finding should make us rethink our strategy when dealing with coronary atherosclerosis in DM patients. The use of ischemic assessments, intracoronary morphological imaging, as well as our treatment modalities need to be fine-tuned to match the specific needs of this patient population, which is clearly quite different than the non-DM population. In this endeavor ischemia is only one, but clearly not the only factor to take into account. Ongoing trials will shed more light into this fascinating model of human atherosclerosis progression.

## Abbreviations

FFR: fractional flow reserve
PCI: percutaneous coronary intervention
CABG: coronary artery by-pass graft
MI: myocardial infarction
DM: diabetes mellitus
TLR: target lesion revascularisation
TLF: target lesion failure
TVMI: target vessel myocardial infarction
CVD: cardiovascular disease
CAD: coronary artery disease
CFR: coronary flow reserve
IMR: index of microvascular resistance
SPECT: single photon emission computed tomography
CT: computerized tomography
MACE: major adverse cardiac events
CAC: coronary artery calcium
NCL: non-culprit lesion
TCFA: thin cap fibroatheroma
